# A Novel Molecule in Human Cyclic Endometrium: LncRNA TUNAR Is Involved in Embryo Implantation

**DOI:** 10.3389/fphys.2020.587448

**Published:** 2020-11-19

**Authors:** Yuan Wang, Shuanggang Hu, Guangxin Yao, Qinling Zhu, Yaqiong He, Yao Lu, Jia Qi, Rui Xu, Ying Ding, Jiaxing Li, Xinyu Li, Yun Sun

**Affiliations:** ^1^Center for Reproductive Medicine, Renji Hospital, School of Medicine, Shanghai Jiao Tong University, Shanghai, China; ^2^Shanghai Key Laboratory for Assisted Reproduction and Reproductive Genetics, Shanghai, China

**Keywords:** long non-coding RNA, TUNAR, endometrial receptivity, embryo implantation, biomarker

## Abstract

Embryo implantation rate remains an inefficient process in *in vitro* fertilization and embryo transfer (IVF-ET) cycles. The role long non-coding RNA (lncRNA) plays in embryo implantation remains unclear. We aimed to investigate the expression pattern of lncRNA TCL1 upstream neural differentiation-associated RNA (TUNAR) in human cyclic endometrium and clarify the role of TUNAR in the development of endometrial receptivity. Endometrial biopsies were collected at the late proliferative phase, luteinizing hormone (LH) + 2 and LH + 7, from patients with or without recurrent implantation failure (RIF). Real-time RT PCR was performed to detect the level of lncRNAs. After pZW1-snoVector-TUNAR transfection, multiple function of TUNAR in endometrial epithelial cells (EECs) and endometrial stromal cells (ESCs) was investigated. The expression of TUNAR in endometrium was found down-regulated at LH + 7 and up-regulated in RIF patients. In proliferative phase, TUNAR was overwhelmingly more abundant in ESCs and regulated its proliferation. In LH + 7, the difference in the expression of TUNAR between ESCs and EECs was narrowed. Overexpression of TUNAR not only impaired spheroid attachment to EECs, but also inhibited decidualization of ESCs. TUNAR was found expressed in human endometrium for the first time, which might be involved in embryo implantation by modulating the blastocyst attachment to the endometrial epithelium and regulating the proliferation and decidualization of ESCs. Our study helps us to better understand the molecular mechanisms of embryo implantation and may provide a promising biomarker of endometrial receptivity.

## Introduction

Despite the technical advances that have continued to evolve in the area of assisted reproduction, overall pregnancy and implantation rates have remained relatively low. It is clear that the success of *in vitro* fertilization and embryo transfer (IVF-ET) cycles not only depends upon embryo quality but also on uterine receptivity as the transfer of euploid embryos following preimplantation genetic testing for aneuploidies (PGT-A) still does not guarantee implantation (De Rycke et al., 2015). The latter is likely to be the result of transferring embryos into a non-receptive uterus (Sharkey and Macklon, 2013).

The process of implantation in the uterus results from a complex set of events as a result of highly orchestrated “cross talk” between a euploid blastocyst and a receptive endometrium. These occur through a series of coordinated genetic and hormonal events regulating intracellular signaling in both the host uterus and implanting blastocyst (Dominguez et al., 2002). When this cross talk fails to occur repeatedly, this phenomenon is referred to as recurrent implantation failure (RIF) in spite of the transfer of normal appearing embryos fails to lead to the stage of recognizable intrauterine sac (Coughlan et al., 2014).

The process of embryo implantation has been widely studied, but our knowledge on specific molecular mechanisms remains incomplete. Many proteins have been verified as endometrial receptivity biomarkers, including homeobox A10 (HOXA10), leukemia inhibitory factor (LIF), and αvβ3 integrin (Aghajanova et al., 2008; Hamamah and Dechaud, 2009). However, only 2% of our genome encodes proteins, while the vast majority is non-coding DNA (Batista and Chang, 2013). Long non-coding RNAs (lncRNAs) are more than 200 nucleotides in length and do not code for proteins (Ponting et al., 2009), and involved in the regulation of diverse biological processes including carcinogenesis, epigenetic regulation, and embryonic development (Wapinski and Chang, 2011; Batista and Chang, 2013). Using high-throughput sequencing analysis, recent studies have attempted to uncover key lncRNAs as biomarkers to predict endometrial receptivity (Koot et al., 2016; Fan et al., 2017; Sigurgeirsson et al., 2017; Chen et al., 2018, 2019; Feng et al., 2018), while little is known about their biological function and significance in the human endometrium. The expression of H19 was down-regulated in the endometrium of RIF (Zeng et al., 2017). LncRNA882 acted as a ceRNA for miR-15b and then indirectly regulated the level of LIF in goat endometrial epithelial cells (EECs) (Zhang et al., 2019).

In our previous study (Hu et al., 2014), a comparison between the transcriptome of luteinizing hormone (LH) + 2 (pre-receptive) and LH + 7 (receptive) human endometrium by RNA sequencing (RNA-seq) identified novel candidate genes, believed to serve as molecular markers for endometrial receptivity. In this study, we explored the function of TCL1 upstream neural differentiation-associated RNA (TUNAR) in the regulation of endometrial receptivity, as this lncRNA came up during our RNA-seq analysis. As is the very first study of TUNAR in human endometrium, we found that it had multiple functions in the endometrium. Not only did TUNAR affect the adhesion of embryos to epithelium, but also regulated the proliferation and decidualization of endometrial stromal cells (ESCs). The process of embryo implantation has been classified into three stages: apposition, adhesion, and penetration, requiring the participation of embryos, EECs and ESCs (Dey et al., 2004). Our study demonstrates the indispensable role of TUNAR in embryo implantation and may provide a promising biomarker of endometrial receptivity.

## Materials and Methods

### Ethics Statement

This study was reviewed and approved by the Ethics Committee of Renji Hospital, Shanghai, China (License No. 2018072608). Written consents were obtained from all participants.

### Participants and Sample Collection

Endometrial tissues were collected from patients who consented to undergo IVF or intra-cytoplasmic sperm injection (ICSI) at the Center for Reproductive Medicine, Renji Hospital, School of Medicine, Shanghai Jiao Tong University.

49 Endometrial samples were obtained by Pipelle catheters in (1) 15 women aged between 20 and 35 years with tubal infertility in a natural cycle prior to undergoing their IVF cycle either on day 10–12 of the proliferative phase, based on the last menstrual period (*n* = 5), at LH + 2 (*n* = 5) and LH + 7 (*n* = 5) (All participants underwent daily urine test from day 9 of the cycle onward to identify the LH surge. If urine LH were found positive, serum LH and progesterone levels were quantified immediately. The day on which serum LH level peaked (≥40 IU/L) and P4 level was less than 1 μg/L was deemed to be the day of LH + 0.) as previously described (Hu et al., 2014); (2) women with tubal infertility following hCG trigger (LH + 7) in a freeze all IVF cycle (*n* = 18); and (3) RIF patients (fail to achieve clinical pregnancy after three or more transfers of high quality embryos) (Polanski et al., 2014) following hCG trigger (LH + 7) in a freeze all IVF cycle (*n* = 16). The inclusion criteria for both groups were as follows: regular ovulatory cycles of 28–32 days, normal body mass index (BMI) (18–24 kg/m^2^), normal endocrine profile including normal serum levels of follicle-stimulating hormone (FSH) (<10 mIU/mL) and LH (< 10 mIU/mL) on day 3 of the menstrual cycle. The exclusion criteria for all participants were: intrauterine pathology (fibroids, polyps, intrauterine adhesions, congenital uterine anomaly), hydrosalpinx, adenomyosis, endometriosis, polycystic ovary syndrome (PCOS), couples with abnormal karyotypes and severe male factor infertility. High quality embryo was defined as having 6–10 blastomeres, uniform size of the blastomeres and fragmentation ≤ 20%. High quality blastocyst was defined as having a grade of at least 3BB, including 3/4/5AA, AB, BA, or BB. All samples were immediately frozen in liquid nitrogen and stored at −80°C for further use.

The basal serum hormonal profiles including FSH, LH, and anti-müllerian hormone (AMH) were determined using chemiluminescence assay kits (Beckman Access Health Co) and an ELISA kit (Kangrun).

### Isolation and Culture of Endometrial Cells

Endometrial cells were enzymatically isolated from human endometrium curettage samples according to a selective attachment method with minor modifications (Kirk et al., 1978). EECs and ESCs from the same patient in the late proliferative phase (*n* = 3) were isolated to compare TUNAR expression, the same method was applied to the ones in the LH + 7 phase (*n* = 3). Others were used for further *in vitro* experiments. All endometrial specimens were minced into small pieces (1 mm^3^) and digested with 0.2% (w/v) collagenase I (Sigma-Aldrich) for 60 min at 37°C, followed by 0.1% (w/v) deoxyribonuclease I (Sigma-Aldrich) for 20 min. Subsequently, tissues were first filtered through a 200-μm (pore size) mesh to remove debris, and then through a 40-μm (pore size) mesh to separate EECs from ESCs. ESCs passed through two strainers and were cultured in phenol red-free DMEM/F12 (GIBCO) supplemented with 10% (v/v) fetal bovine serum (FBS) (BI) at 37°C and 5% CO_2_. EECs were retained on the 40-μm sieve and were collected by backwashing the 40-μm filter sieve and resuspended in phenol red-free DMEM/F12 containing 10% FBS. Cell purity was tested routinely by immunofluorescence staining for cytokeratin 7 (CK7) and vimentin. Culture medium was changed every other day.

### Immunofluorescence Staining

On the second day of culture, the cells in the chamber slide (BD Biosciences) were fixed with 4% paraformaldehyde and permeabilized with 0.4% Triton X-100. After blocking, the cells were incubated with antibodies against CK7 (1:200; Proteintech, United States) and vimentin (1:200; Proteintech, United States) overnight at 4°C. After washing, Alexa Fluor 594 goat anti-mouse immunoglobulin G (red; 1:200; Proteintech) and Alexa Fluor 488 goat anti-rabbit immunoglobulin G (green; 1:200; Proteintech) were used as secondary antibodies and the cells were incubated in darkness for 1 h. Nuclei were stained with 4′,6′-diamino-2-phenylindole (DAPI) (1 mg/mL). Images were obtained with a microscope and camera connected to a computer with an image analysis system (Zeiss).

### Subcellular Fractionation

Cytoplasmic and nuclear RNA in human ESCs (*n* = 3) and EECs (*n* = 3) was isolated and collected using a Cytoplasmic and Nuclear RNA Purification Kit (Norgen Biotek) following the protocol provided by the manufacturer.

### Treatment of Estradiol and Progesterone

Cells were plated in 6-well plates (5 × 10^5^ cells/well) (Corning) and cultured in phenol red-free DMEM/F12 supplemented with 10% charcoal-stripped FBS at 37°C and 5% CO_2_ for 24 h. After starvation in serum-free and phenol red-free DMEM/F12 for 24 h, the cells were treated with 17β-estradiol (E2) (0.0001, 0.001, 0.01 μM) (Sigma) and progesterone (P4) (0.01, 0.1, 1 μM) (Sigma) in phenol red-free DMEM/F12 supplemented with 10% charcoal-stripped FBS for 24 h. The same concentration of dimethyl sulfoxide (DMSO) was used in the control groups.

### Plasmid Construction and Transfection

For the construction of a plasmid expressing TUNAR, TUNAR cDNA was amplified by PCR with the following primers: forward, 5′-GTCAAGCTTGTGCAGCTCTGCGCGTTCTCATG-3′ and reverse, 5′-GTCGGTACCCAGAGCCTACTGAGTAGCTCCTT CC-3′. Then, the sequence of TUNAR was inserted between the *Kpn*I site and *Hin*dIII site of the pZW1-snoVector (from Dr. Ling-Ling Chen, Chinese Academy of Sciences) (Figure 3C; Yin et al., 2015). The sequence of the TUNAR overexpression plasmid (pZW1-snoVector-TUNAR) was confirmed (Majorbio, China). ESCs were seeded into a 6-well plate and cultured in DMEM/F12 supplemented with 10% FBS. When the cell density reached 70–80%, ESCs were transfected with pZW1-snoVector-TUNAR or the control plasmid pZW1-snoVector using Lipofectamine^®^ 3000. The medium was replaced 6 h after the transfection and the total RNA was harvested at 48 h post-transfection. The overexpression efficiency was verified by real-time RT PCR.

### *In vitro* Embryo Implantation

*In vitro* embryo implantation study was conducted as described (Liu et al., 2010; Tsang et al., 2012) with modifications. Ishikawa cell (a human endometrial adenocarcinoma cell line) was a gift from Prof. Huang HF (International Peace Maternity and Child Health Hospital, Shanghai, China). Human endometrial epithelial cell line (HEEC) (derived from human primary cells) was purchased from iCell Bioscience Inc. (Shanghai, China). JAR cell (a human choriocarcinoma cell line) was purchased from the Cell bank of the Chinese academy of sciences (Shanghai, China). Ishikawa cells or HEEC were seeded into 12-well plates at density of 5 × 10^5^ cells/well and incubated in DMEM/F12 supplemented with 10% FBS at 37°C in a 5% CO_2_-humidified incubator. After reaching 80% confluence, Ishikawa cells or HEEC were transfected with pZW1-snoVector-TUNAR or the control plasmid pZW1-snoVector using Lipofectamine^®^ 3000. Attachment assay was conducted 48 h after transfection. JAR cells were seeded into 6-well plates at density of 3 × 10^5^ cells/well, and were incubated in RPMI1640 supplemented with 10% FBS, 1% antibiotics and antimycotics (100×; Thermo Fisher Scientific, Inc.) for 24 h on a shaker at 108 rpm at 37°C in a 5% CO_2_–humidified incubator. Thirty spheroids (100–200 μm in diameter) were transferred per well onto the confluent monolayer of Ishikawa cells. After incubation with the spheroids for 1 h, the attached spheroids were counted, and the attachment rate was expressed as a percentage of the total number of spheroids (% adhesion).

### BrdU Cell Proliferation Assay

ESCs were plated in 6-well plates at a density of 3 × 10^5^ cells/well and incubated overnight. After ESCs were transfected with pZW1-snoVector-TUNAR or control plasmid, 5-bromodeoxyuridine (BrdU) was added into the cells to reach a final concentration of 100 μM and incubated for 2 h in an incubator at 37°C with 5% CO_2_. After the medium was removed, the cells were fixed by 4% paraformaldehyde for 15 min and then permeabilized with 0.4% Triton X-100. After washing, 2 N HCL was added for 30 min and then 0.1 M NaB_4_O_3_ for 15 min at room temperature. The solution was removed and the cells were blocked with goat serum for 1 h and then incubated with antibodies against BrdU (1:1,000; Cell Signaling Technology) overnight at 4°C. Alexa Fluor 594 goat anti-mouse immunoglobulin G (red; 1:200; Proteintech) was used as secondary antibodies and incubated in darkness for 1 h. The nuclei were counterstained with DAPI (blue). The staining was examined using a fluorescence microscope (Zeiss).

### *In vitro* Decidualization

To induce *in vitro* decidualization, the ESCs were incubated with phenol red-free DMEM/F12 containing 2% stripped FBS, 5 × 10^–4^ M N6, 2′-O-dibutyryladenosine cAMP sodium salt (cAMP) (Sigma Chemical Co., St. Louis, MO, United States) and 10^–6^ M medroxyprogesterone-17-acetate (MPA) (Sigma Chemical Co., St. Louis, MO, United States) for 0–8 days. To investigate the effect of TUNAR on decidualization, ESCs were plated in medium lacking antibiotics at approximately 3 × 10^5^ cells/well in 6-well plates. The pZW1-snoVector-TUNAR or the control plasmid pZW1-snoVector diluted in Opti-MEM Medium (GIBCO) were transfected to ESCs at 70% confluence. After 24 h of transfection, the ESCs were incubated with medium in the presence or absence of cAMP and MPA for 4 days.

### RNA Extraction and Real-Time RT PCR

Tissue and cellular total RNA was extracted using a total RNA kit (Foregene, China). A NanoDrop^®^ ND-2000 spectrophotometer (Thermo Fisher Scientific, United States) was used to measure the quantity and quality of total RNA. RNA was reverse-transcribed to cDNA using PrimeScript^®^ RT Master Mix Perfect Real Time kit (TaKaRa, China). Real-time RT PCR was performed using SYBR Premix Ex Taq (TaKaRa, China) and signals were detected with ABI7500 Real-Time PCR System (Applied BioSystems, United States). The ratio of the target gene to ACTB was obtained to normalize the expression of the target gene. All experiments were carried out in triplicate. The primer sequences used are given in Supplementary Table 1.

### Western Blot

Total protein was extracted using ice-cold radioimmunoprecipitation assay (RIPA) lysis buffer (Cwbio, China) containing a phosphatase inhibitor (Roche, Switzerland) and a protease inhibitor cocktail (Roche). After the protein was separated by 10% SDS-PAGE, the wet transfer method was used to transfer the isolated proteins to nitrocellulose blot (Millipore, Germany) at 200 mA for 1 h. After 1 h blocking, the blot was incubated overnight at 4°C with antibodies against proliferating cell nuclear antigen (PCNA) (1:1,000; Santa Cruz, United States). On the second day, the blot was washed and then incubated with the respective secondary antibody conjugated to horseradish peroxidase (Proteintech, United States) for 1 h at room temperature. A G-Box iChemi Chemiluminescence image capture system (Syngene, United States) was used to visualize the bands. The same blot was also probed with actin (1:10,000; Proteintech, United States) as internal controls. The experiments were repeated at least three times.

### Statistical Analysis

Data are presented as mean ± SEM of at least three independent experiments. Analyses were performed using the Statistical Package for Social Science (version 20.0; SPSS) and Graphpad Prism statistical software (version 5.0, Graphpad). After evaluating the normal distribution assumption using the Kolmogorov-Smirnov test, unpaired *t*-test and one-way ANOVA followed by Student–Newman–Keuls test were performed when appropriate. Spearman correlation was used to analyze the relation between variables. A value of *P* < 0.05 was considered as statistically significance.

## Results

### The Cyclic Expression of TUNAR in Endometrium From Healthy Women and Its Abnormal Expression in RIF Patients

**FIGURE 1:**
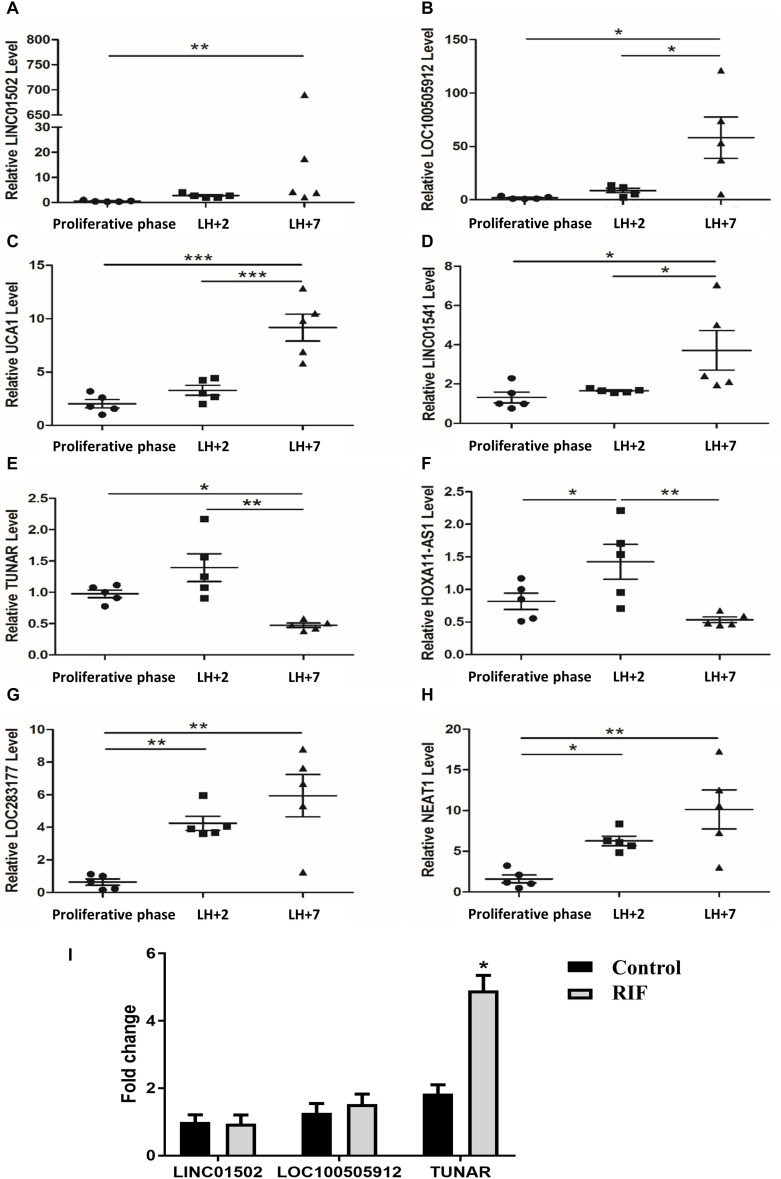
The expression of lncRNAs in the endometrium. The expression of **(A)** LINC01502, **(B)** LOC100505912, **(C)** UCA1, **(D)** LINC01541, **(E)** TUNAR, **(F)** HOXA11-AS1, **(G)** LOC283177, **(H)** NEAT1 in late proliferative phase (*n* = 5), LH + 2 (*n* = 5), and LH + 7 phase (*n* = 5) in human endometrium. **(I)** The expression of LINC01502, LOC100505912, and TUNAR in LH + 7 endometrium from RIF patients (*n* = 16) and controls (*n* = 18). *P < 0.05, **P < 0.01, ***P < 0.001.

RNA-seq was performed in our previous work (Hu et al., 2014) to compare the transcriptome profiles between LH + 2 and LH + 7 endometrium. Eight lncRNAs showed differential expression with a log2| FC| > 1.5, *P* < 0.001 (Supplementary Table 2). We analyzed the expression levels of the eight lncRNAs in human cyclic endometrium by real-time RT PCR. The endometrium samples were collected at three different time points during the menstrual cycle (the late proliferative phase, LH + 2, and LH + 7). The levels of LINC01502 (Figure 1A), LOC100505912 (Figure 1B), urothelial cancer associated 1 (UCA1) (Figure 1C), and LINC01541 (Figure 1D) were significantly increased in LH + 7 compared with those in the late proliferative phase and LH + 2. The expression of TUNAR (Figure 1E) and HOXA11 antisense RNA (HOXA11-AS1) (Figure 1F) was highest in LH + 2 endometrium, while it was lowest in LH + 7 samples. The expression of LOC283177 (Figure 1G) and nuclear paraspeckle assembly transcript 1 (NEAT1) (Figure 1H) in the endometrium was gradually increased from the proliferative phase to LH + 7. These results are consistent with our previous RNA-seq data (Supplementary Table 2), suggesting that these eight lncRNAs might be involved in endometrial receptivity.

We further explored the expression of the top three lncRNAs with highest log2 | FC| in the LH + 7 endometrium from RIF patients. Supplementary Table 3 summarized the demographic characteristics of RIF (*n* = 16) and controls (*n* = 18). Age, BMI, basal FSH, basal LH, AMH, number of embryo per transfer were comparable. It was shown that TUNAR expression was significantly increased in RIF patients compared with controls, while the expression of LINC01502 and LOC100505912 was similar between these two groups (Figure 1I). Due to the cyclic expression in human endometrium and its abnormal expression in RIF patients, TUNAR may participate in regulating the process of embryo implantation.

### The Localization of TUNAR in Human Endometrium

**FIGURE 2:**
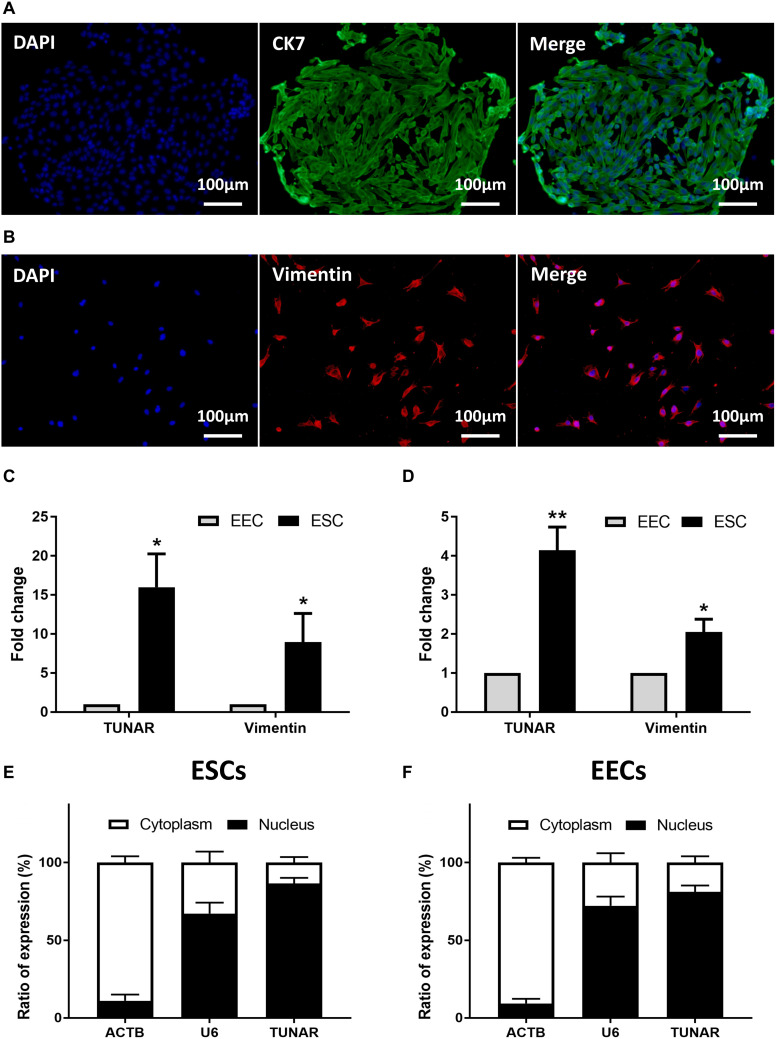
Localization of TUNAR in human endometrium. Immunofluorescence staining of CK 7 (green) in EECs **(A)** and vimentin (red) in ESCs **(B)** (200×). The nuclei were stained with 4′,6-diamidino-2-phenylindole (DAPI) (blue). **(C)** The expression of vimentin mRNA and TUNAR in EECs and ESCs from the same patients in the late proliferative phase (*n* = 3). **(D)** The expression of vimentin mRNA and TUNAR in EECs and ESCs from the same patients in LH + 7 phase (*n* = 3). The subcellular localization of ACTB, U6, and TUNAR in **(E)** ESCs (*n* = 3) and **(F)** EECs (*n* = 3). *P < 0.05, **P < 0.01.

The isolated human primary EECs (Figure 2A) and ESCs (Figure 2B) were identified by immunofluorescence staining with CK 7 (marker of epithelial cells) and vimentin (marker of stroma cells). We further compared TUNAR abundance in EECs and ESCs to determine its cellular expression pattern. In samples from the late proliferative phase, TUNAR was overwhelmingly more abundant in ESCs than EECs (Figure 2C). In LH + 7 phase, the expression of TUNAR was significantly higher in ESCs (Figure 2D), but the difference between these two cells was narrowed compared to the late proliferative phase. Vimentin was used to demonstrate the purity of cells. In addition, we examined the subcellular localization of TUNAR. The nuclear and cytoplasmic RNA were separated, and it was found that TUNAR located predominantly in the nuclei in both ESCs (Figure 2E) and EECs (Figure 2F). ACTB and U6 were used as cytoplasm and nucleus controls, respectively.

**FIGURE 3:**
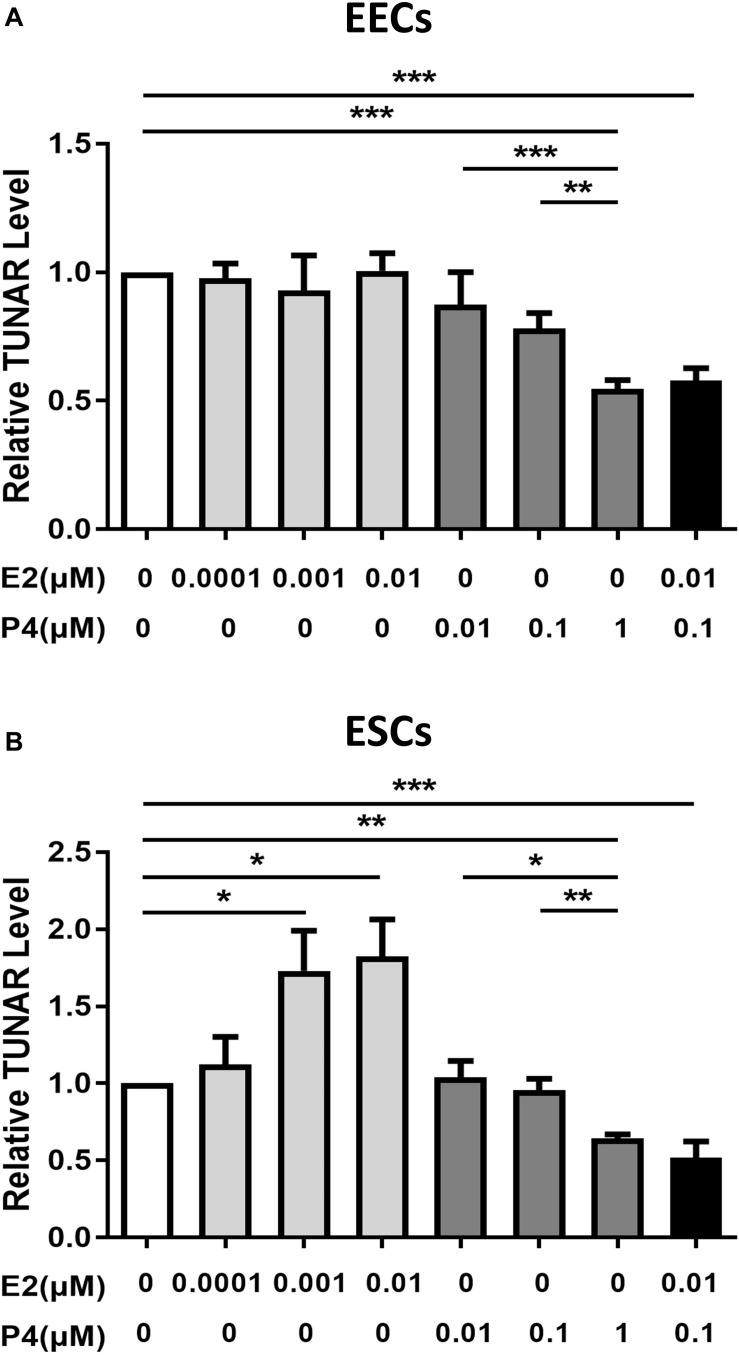
The effect of E2 and P4 on TUNAR expression in human endometrium. Effects of E2 (0, 0.0001, 0.001, 0.01 μM, 24 h), P4 (0, 0.01, 0.1, 1 μM, 24 h) and E2 (0.01 μM) combined with P4 (0.1 μM) on TUNAR expression in **(A)** EECs (*n* = 3) and **(B)** ESCs (*n* = 3). *P < 0.05, **P < 0.01, ***P < 0.001.

### Effect of Estradiol and Progesterone on the Expression of TUNAR

E2 and P4, the principal hormones that regulate uterine function, were added to explore their effects on the expression of TUNAR in EECs and ESCs. In EECs (Figure 3A), P4 inhibited the expression of TUNAR in a dose-dependent manner, while E2 didn’t affect TUNAR expression. When P4 (0.1 μM) and E2 (0.01 μM) were added together, TUNAR expression was significantly inhibited. In ESCs (Figure 3B), E2 promoted the expression of TUNAR at concentration of 0.0001, 0.001, 0.01 μM, while P4 inhibited it at 0.01, 0.1, 1 μM in a dose-dependent manner. When combined with P4 (0.1 μM) and E2 (0.01 μM), TUNAR expression was significantly down-regulated.

### TUNAR Overexpression in Human endometrial Epithelial Cells Impaired JAR Spheroids Attachment in *in vitro* Implantation Model

**FIGURE 4:**
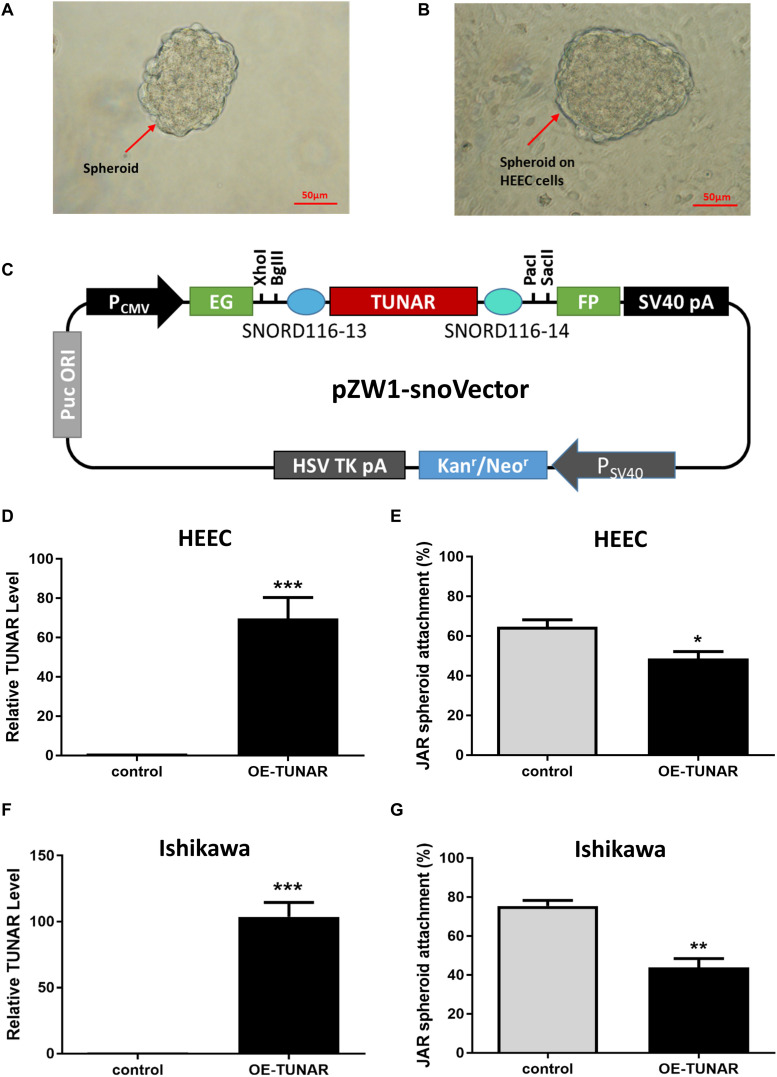
Effect of TUNAR on human embryo implantation. **(A)** JAR cells were trypsinized and shaked for 24 h to obtain spheroids of 100–200 μm in size. **(B)** The spheroids were put onto an human endometrial epithelial cell line (HEEC) monolayer for an hour and the number of spheroids attached was determined as a percentage of the number of spheroids added. **(C)** A schematic view of the pZW1-snoVector-TUNAR. **(D)** Expression of TUNAR in HEEC after TUNAR overexpression for 48 h (*n* = 3). **(E)** Changes in JAR spheroid attachment rate (attach to HEEC) after TUNAR overexpression for 48 h (*n* = 3). **(F)** Expression of TUNAR in Ishikawa cells after TUNAR overexpression for 48 h (*n* = 3). **(G)** Changes in JAR spheroid attachment rate (attach to Ishikawa) after TUNAR overexpression for 48 h (*n* = 3). *P < 0.05, **P < 0.01, ***P < 0.001.

To assess whether TUNAR have an effect on the blastocyst attachment to the endometrium, we performed the attachment assay. JAR spheroids (Figure 4A) served as embryonic bodies were added onto a single layer of human endometrial epithelial cells, and coculture was maintained for 1 h (Figure 4B). As mentioned before, TUNAR was constrained to the nucleus. So the pZW1-snoVector-TUNAR plasmid (Figure 4C) was used to stably express TUNAR and constrain its accumulation to the nucleus to exert its functions (Yin et al., 2015). Spheroid attachment was significantly inhibited when human endometrial epithelial cells (HEEC and Ishikawa) were transfected by TUNAR overexpression plasmid (Figures 4D–G). These data suggest that TUNAR does play an important role in embryo attachment *in vitro*.

### Effect of TUNAR on Proliferation and Decidualization of ESCs

**FIGURE 5:**
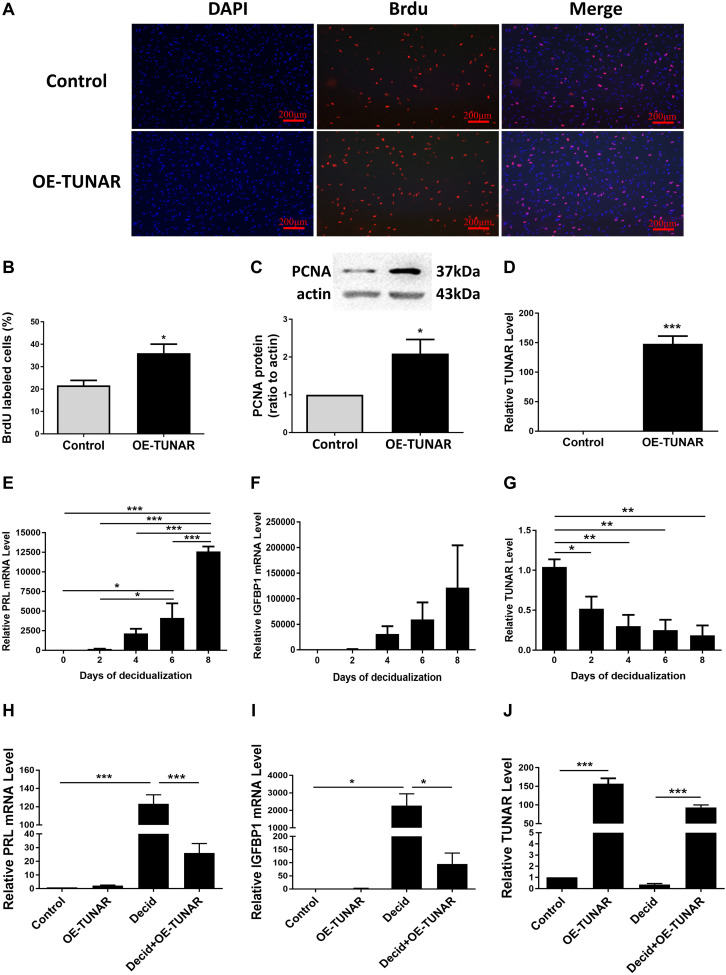
Effect of TUNAR on ESCs proliferation and its function during decidualization. **(A,B)** BrdU cell proliferation assay. The pictures **(A)** and numbers **(B)** of the BrdU positive cells were acquired under the fluorescence microscope (100×). BrdU (red) was incorporated into ESCs during S phase. Nuclei were stained with 4′,6-diamidino-2-phenylindole (DAPI; blue). **(C)** PCNA protein levels in ESCs transfected with either TUNAR overexpression vector (OE-TUNAR) or negative control vector (Control); protein samples were collected 48 h after transfection. **(D)** Expression of TUNAR in ESCs 48 h after transfection with either TUNAR overexpression vector (OE-TUNAR) or negative control vector (Control). The ESCs were treated for 0, 2, 4, 6, 8 days, respectively, then analyzed the mRNA levels of PRL **(E)**, IGFBP1 **(F)**, and level of TUNAR **(G)** by real-time RT-PCR. Overexpression of TUNAR decreases PRL **(H)** and IGFBP1 **(I)** expression in decidualized ESCs. **(J)** TUNAR expression after transfected with plasmids. Data are representative of at least three independent experiments. *P < 0.05, **P < 0.01, ***P < 0.001.

ESCs separated from the late proliferative phase were cultured *in vitro*. The BrdU cell proliferation assay indicated that TUNAR overexpression stimulated the proliferation ability of the ESCs (Figures 5A,B). In addition, the abundance of PCNA protein was significantly increased in the TUNAR-overexpressed ESCs (Figure 5C). The efficiency of the vector transfection was confirmed by real-time RT-PCR (Figure 5D). These data together pointed out that TUNAR overexpression stimulated the growth of ESCs in the proliferative phase.

To explore the expression of TUNAR during decidualization, we induced decidualization of ESCs for 0, 2, 4, 6, and 8 days, respectively. The levels of decidualization markers prolactin (PRL) and insulin like growth factor binding protein 1 (IGFBP1) mRNA increased significantly in a time-dependent manner (Figures 5E,F). Corresponding to the decidualization stimulation, the expression of TUNAR significantly decreased (Figure 5G). Furthermore, TUNAR overexpression led to significant reductions in the mRNA levels of both PRL and IGFBP1 in decidualized ESCs (Figures 5H–J). These results indicated the importance of TUNAR during decidualization.

## Discussion

To our knowledge, we are the first one to explore the expression pattern of lncRNA TUNAR in human cyclic endometrium and its function in embryo implantation. We demonstrated that TUNAR is expressed in human cyclic endometrium with its expression levels increased at LH + 2 and decreased at LH + 7. In addition, the expression of TUNAR increased in the LH + 7 endometrium from RIF patients. Overexpression of TUNAR in Ishikawa or HEEC cells significantly inhibited the JAR spheroids attachment in the *in vitro* embryo implantation model. Moreover, overexpression of TUNAR stimulated the proliferation of ESCs and inhibited decidualization.

TUNAR, also known as linc00617, was identified in the comparison between the transcriptome of LH + 2 and LH + 7 human endometrium by our previous RNA-seq (Hu et al., 2014). It is evolutionarily conserved and its orthologous gene, TUNA, is required for the maintenance of pluripotency of mouse embryonic stem cells (Lin et al., 2014). Moreover, it has been reported that TUNAR functions as an important regulator of epithelial-mesenchymal transition (EMT) and promotes breast cancer progression and metastasis (Li et al., 2017). In addition, TUNAR has been detected in metaphase II (MII) oocytes (Bouckenheimer et al., 2018) and prostate cancer tissue (Ramnarine et al., 2018). But till now, its function in human endometrium has not been detected. In our study, we observed that TUNAR participates in endometrial receptivity during embryo implantation. During the window of implantation (WOI), the expression of TUNAR was found to be significantly decreased. Besides, TUNAR expression in the endometrium of RIF patients was significantly higher than that of the control patients. We speculate that high concentration of TUNAR may inhibit the expression of implantation-related genes, and down-regulation of TUNAR expression during the WOI may help establish endometrial receptivity.

So, what role does TUNAR play in the establishment of endometrial receptivity? Embryo implantation requires three steps: apposition, adhesion, and penetration. In the first two processes, the embryo is only in direct contact with the EECs. The stage of penetration involves the invasion of the luminal epithelium by the trophectoderm. During the same period, ESCs differentiation into decidual cells (decidualization) is more extensive (Dey et al., 2004; Ashary et al., 2018). Therefore, both EECs and ESCs are indispensable for embryo implantation. Although TUNAR expression was significantly higher in ESCs in LH + 7 phase, the difference between ESCs and EECs was greatly narrowed compared to the late proliferative phase, suggesting that TUNAR may play an essential part both in EECs and ESCs. Models employing trophoblast spheroids have been widely used to study embryo attachment with various human endometrial epithelial cell-like cell lines, such as Ishikawa, RL95-2 and ECC-1 (Weimar et al., 2013). We used the *in vitro* embryo implantation model and found that TUNAR could affect adhesion between embryos and EECs. On the other hand, abnormally increased expression of TUNAR impaired decidualization. The decidua provides a source of growth factors and cytokines that regulate embryo invasion (Ramathal et al., 2010). During the proliferative phase, the proliferative response originates in the stroma and feedbacks growth pathways via paracrine signaling in the endometrial epithelium (Makieva et al., 2018). The increasing mitotic activity seen throughout the endometrial epithelium and stroma intends to thicken the functional layer in preparation for implantation. In the current study, TUNAR was found overwhelmingly more abundant in ESCs than EECs in endometrium at the late proliferative phase. And overexpression of TUNAR resulted in a significant increase in the proliferation ability of ESCs. These indicate that TUNAR participates in the establishment of endometrial receptivity by affecting the function of EECs and ESCs during WOI and regulates the growth of ESCs before ovulation.

Finally, during the WOI, the endometrium undergoes extensive morphological and physiological changes, which is precisely regulated, to facilitate embryo implantation. Among all the regulating elements, ovarian steroids are the leading factors (Paulson, 2011; Cha et al., 2012). It has been reported that many important genes related to endometrial receptivity were regulated by estrogen and progesterone, for example, progesterone receptor (PR), Indian hedgehog (IHH), LIF, etc. (Cha et al., 2012). We found that TUNAR was also regulated in the endometrium by estrogen and progesterone. This response explained the significant decrease of TUNAR in LH + 7 endometrium and also suggested a correlation between TUNAR and endometrial receptivity.

In terms of the important function of TUNAR in the endometrium, we speculate that it may have the potential clinical application as following. At present, before embryo transfer in RIF patients, we often do some pretreatments, such as GnRHa, in order to improve endometrial receptivity and increase clinical pregnancy rate. It may be helpful to assess the endometrial receptivity by comparing the expression of TUNAR in the endometrium of RIF patients before and after the pretreatment, which is expected to help determine the appropriate timing of embryo transfer. In addition, there is still no generally accepted gold standard for evaluating endometrial receptivity. We suppose that TUNAR may be integrated with ERA (Díaz-Gimeno et al., 2011, 2013), which is based on the expression of 238 endometrial genes, to improve the accuracy of the assessment.

## Conclusion

In conclusion, this study provides the first analysis of lncRNA TUNAR in human cyclic endometrium. It might be involved in embryo implantation by modulating the blastocyst attachment to the endometrial epithelium and regulating the proliferation and decidualization of ESCs. Due to the importance of TUNAR in human embryo implantation, further studies are needed to elucidate the underlying mechanism of TUNAR in endometrial receptivity to expedite the clinical use of this potential biomarker.

## Data Availability Statement

All datasets generated for this study are included in the article/Supplementary Material, further inquiries can be directed to the corresponding author.

## Ethics Statement

The studies involving human participants were reviewed and approved by the Ethics Committee of Renji Hospital, Shanghai, China. The patients/participants provided their written informed consent to participate in this study.

## Author Contributions

YS and SH designed the experiments. YW and GY performed the experiments. YL, JQ, RX, YD, JL, and XL collected the clinical samples. QZ and YH analyzed and made interpretation of the data. YW wrote the manuscript. YS, SH, and RX revised it. All authors read and approved the manuscript.

## Conflict of Interest

The authors declare that the research was conducted in the absence of any commercial or financial relationships that could be construed as a potential conflict of interest.
