# Supplementary material for: A Novel Molecule in Human Cyclic Endometrium: LncRNA TUNAR Is Involved in Embryo Implantation
Source: Front Physiol. 2020 Nov 19;11:587448. doi: 10.3389/fphys.2020.587448 (PMC7710794; doi:10.3389/fphys.2020.587448)
Supplement: Supplementary file 1 [file Table_1.docx]

**Supplemental Table I. Primer sequences used for real-time RT PCR.**

| Genes | Forward (5’-3’) | Reverse (5’-3’) |
| --- | --- | --- |
| ACTB | CATGTACGTTGCTATCCAGGC | CTCCTTAATGTCACGCACGAT |
| U6 | CTCGCTTCGGCAGCACA | AACGCTTCACGAATTTGCGT |
| TUNAR | GTGAAAATGATGAAGACAGAGGAGG | AGGACTTCCGTCTTTGGCCA |
| LINC01541 | ATGAGACCTCTCCTCGTCTGTGC | GACTCTGAATCTGCTGGCTCCTTG |
| LINC01502 | CACGATGCAGCCACTAATGC | GTTACCCTGGAAACGCTGGA |
| UCA1 | ATACGGACATGCTTGACACTTGGT | TTCAGCCACTAAGCCGAGGAGAT |
| NEAT1 | GGTGGAGGAGTCAGGAGGAATAGG | TGCTGGCATGGACAAGTTGAAGATT |
| LOC283177 | TCTGGTGGTGAATCCTCCGTAG | AGGCTGAGGCGTAAGAATTGC |
| LOC100505912 | GTTGGCCCGTAGAGTGAACA | CAGCTTGTCTGTCCGTGGAA |
| HOXA11-AS1 | TTTAGAGGCGCTGACATCCG | AGTTTTCTGGAGATGGCCCG |
| Vimentin | GACGCCATCAACACCGAGTT | CTTTGTCGTTGGTTAGCTGGT |
| PRL | CATATTGCGATCCTGGAATGAG | GATGAACCTGGCTGACTATCA |
| IGFBP1 | GGCACAGGAGACATCAGGAGAA | GGTAGACGCACCAGCAGAGT |

Abbreviations: TUNAR: TCL1 upstream neural differentiation-associated RNA; UCA1: urothelial cancer associated 1; NEAT1: nuclear paraspeckle assembly transcript 1; HOXA11-AS1: HOXA11 antisense RNA; PRL: prolactin; IGFBP1: insulin like growth factor binding protein 1.

**Supplemental Table II. SDE lncRNAs with the highest fold change in tissue.**

| Gene | Log2 FC | *P*-value |
| --- | --- | --- |
| LINC01502 | 1.8E+308 | 5.68E−07 |
| LOC100505912 | 4.80 | 7.66E−05 |
| TUNAR | −3.84 | 0 |
| LOC283177 | 3.45 | 1.45E−06 |
| UCA1 | 3.09 | 3.67E−08 |
| NEAT1 | 2.70 | 1.28E−12 |
| LINC01541 | 2.48 | 2.29E−09 |
| HOXA11-AS1 | −1.71 | 9.02E−06 |

Abbreviations: TUNAR: TCL1 upstream neural differentiation-associated RNA; UCA1: urothelial cancer associated 1; NEAT1: nuclear paraspeckle assembly transcript 1; HOXA11-AS1: HOXA11 antisense RNA.

**Supplemental Table III. Demographic characteristics of RIF patients and controls.**

|  | RIF (n = 16) | Controls (n = 18) | P-value |
| --- | --- | --- | --- |
| Age (y) | 31.06±2.29 | 29.94±3.21 | NS |
| BMI (kg/m^2^) | 21.18±1.74 | 20.72±1.52 | NS |
| Basal FSH (mIU/mL) | 6.43±1.66 | 6.38±1.50 | NS |
| Basal LH (mIU/mL) | 5.04±1.33 | 5.27±1.36 | NS |
| AMH (ng/ml) | 3.37±1.02 | 3.97±1.23 | NS |
| Number of embryo per transfer | 1.88±0.34 | 1.72±0.46 | NS |

Data are presented as mean ± SD. Abbreviations: RIF, recurrent implantation failure; BMI: body mass index; FSH: follicle-stimulating hormone; LH: luteinizing hormone; AMH: anti-Müllerian hormone; NS, not significant.
